# Supplementary material for: Regulation Mechanism of Dopamine Receptor 1 in Low Temperature Response of Marsupenaeus japonicus
Source: Int J Mol Sci. 2023 Oct 17;24(20):15278. doi: 10.3390/ijms242015278 (PMC10607110; doi:10.3390/ijms242015278)
Supplement: Supplementary file 1 [file ijms-24-15278-s001.zip › ijms-2608856-supplementary.pdf]

Figure S1

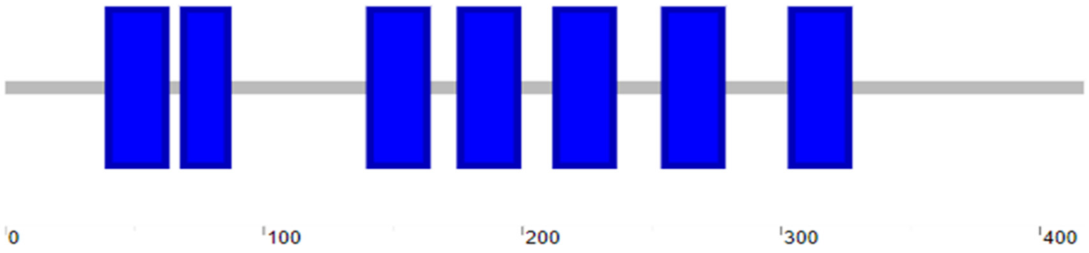

Figure S1 Secondary structure analysis of *MjDRD1*gene from *M. japonicus.*

Figure S2

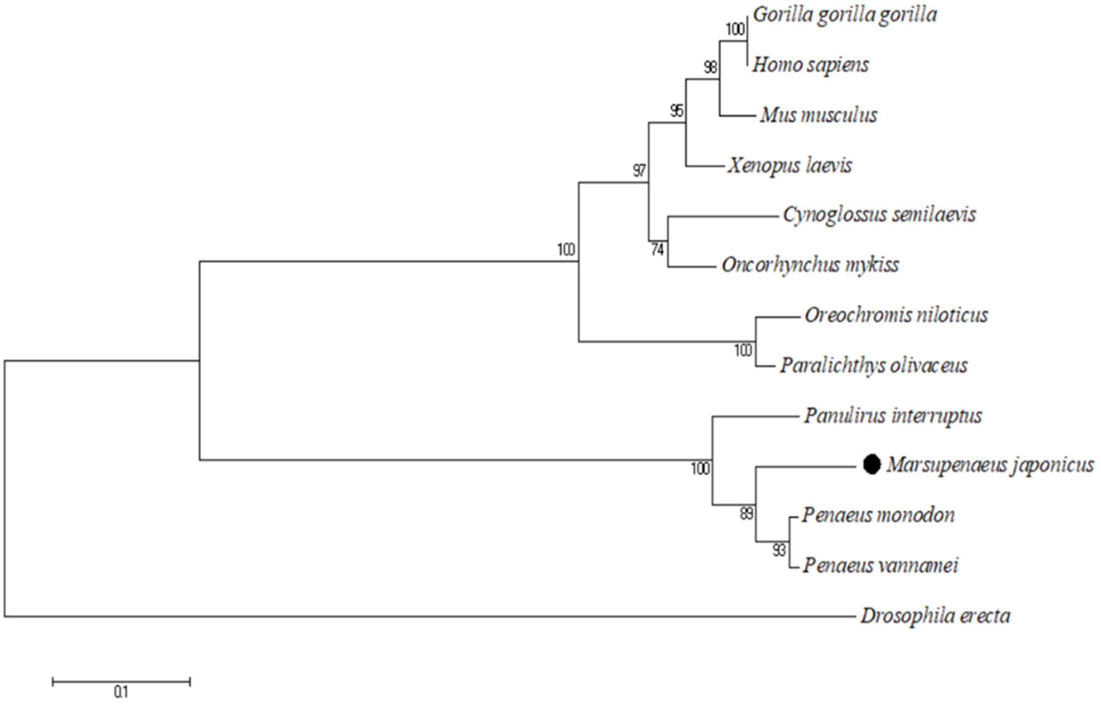

Figure S2 NJ phylogenetic tree of the DAD1 amino acid sequence of *M. japonicus*.

Note：The numbers at the forks indicate the bootstrap proportions.

Name of each species and GenBank accession numbers: *G. gorilla gorilla* XP_018881732.1; *H. sapiens* AAB26273.1; *M. musculus* NP_034206.1; *X. laevis* XP_041442143.1; *C. semilaevis* XP_024918977.1; *O. mykiss* XP_021479832.2; *O. niloticus* XP_025766845.1; *P. olivaceus* XP_019964935.1 ; *P. interruptus* ABB87183.1; *P. monodon* AFX71574.1; *L. vannamei* ROT72626.1; *D. erecta* XP_001978491.1

Figure S3

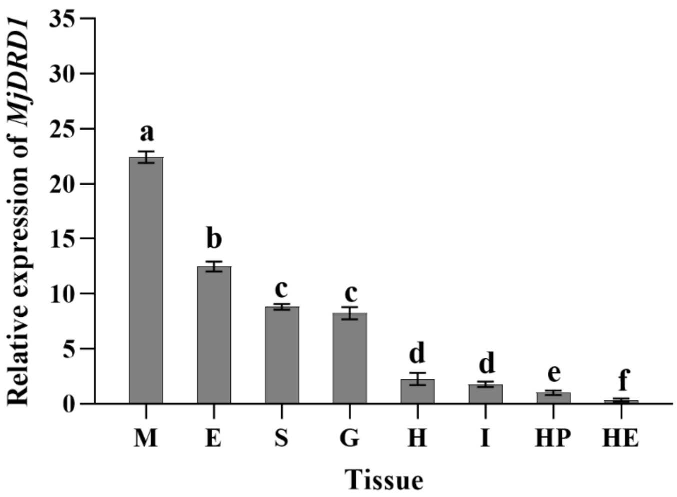

Figure. S3 Distribution of *MjDRD1* gene expression in different tissues of *M. japonicus.* HP: hepatopancreas; M: muscle; H: heart; G: gill; S: stomach; HE: hemocyte; I: intestines; E: eyestalk

Different letters on the column represent significant differences in different tissues ($P < 0.05$).

Figure S4

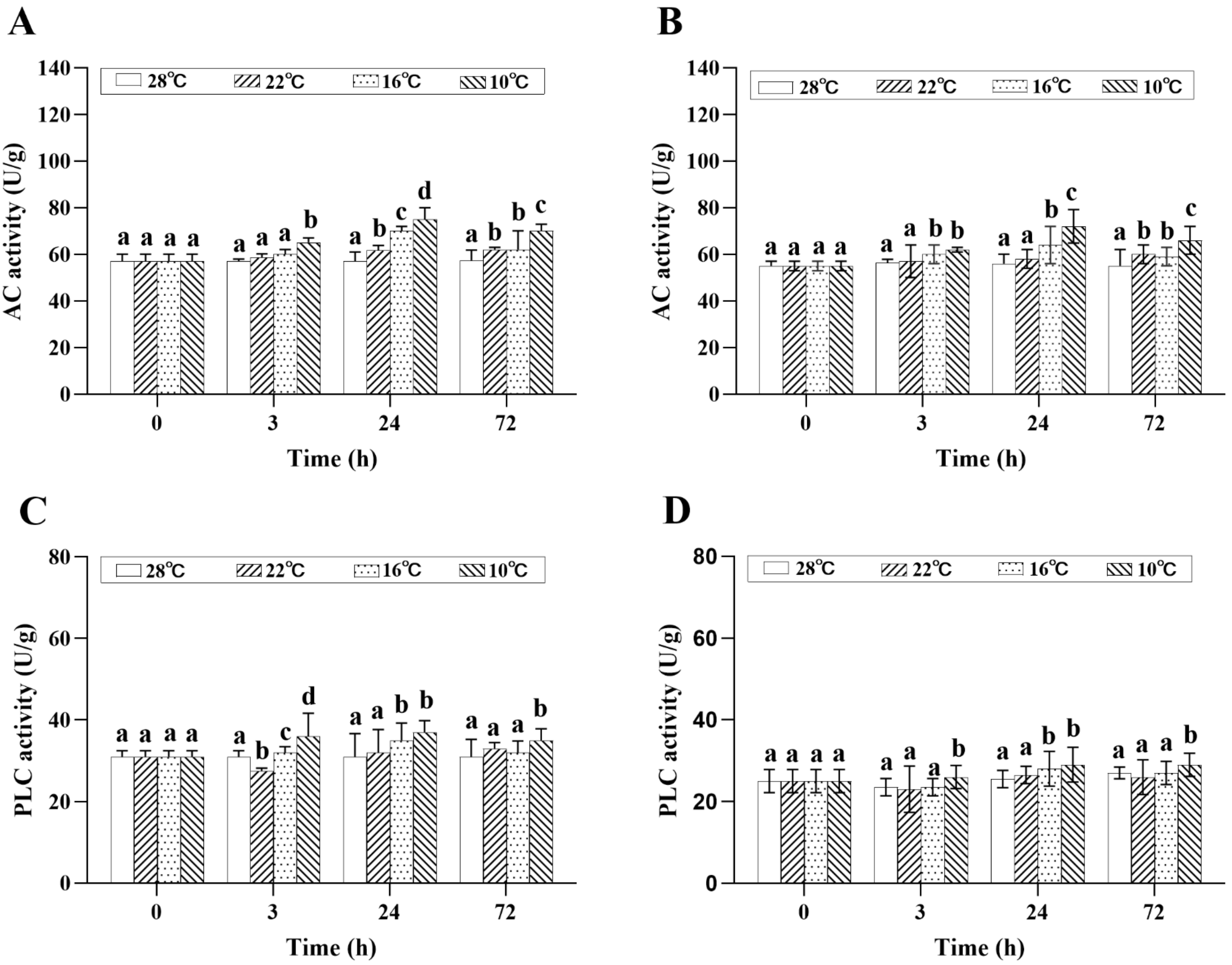

Figure. S4 Changes to AC and PLC levels in the gill (A, C) and hepatopancreas (B, D) of *M. japonicus* according to the time of low temperature stress.

Figure S5

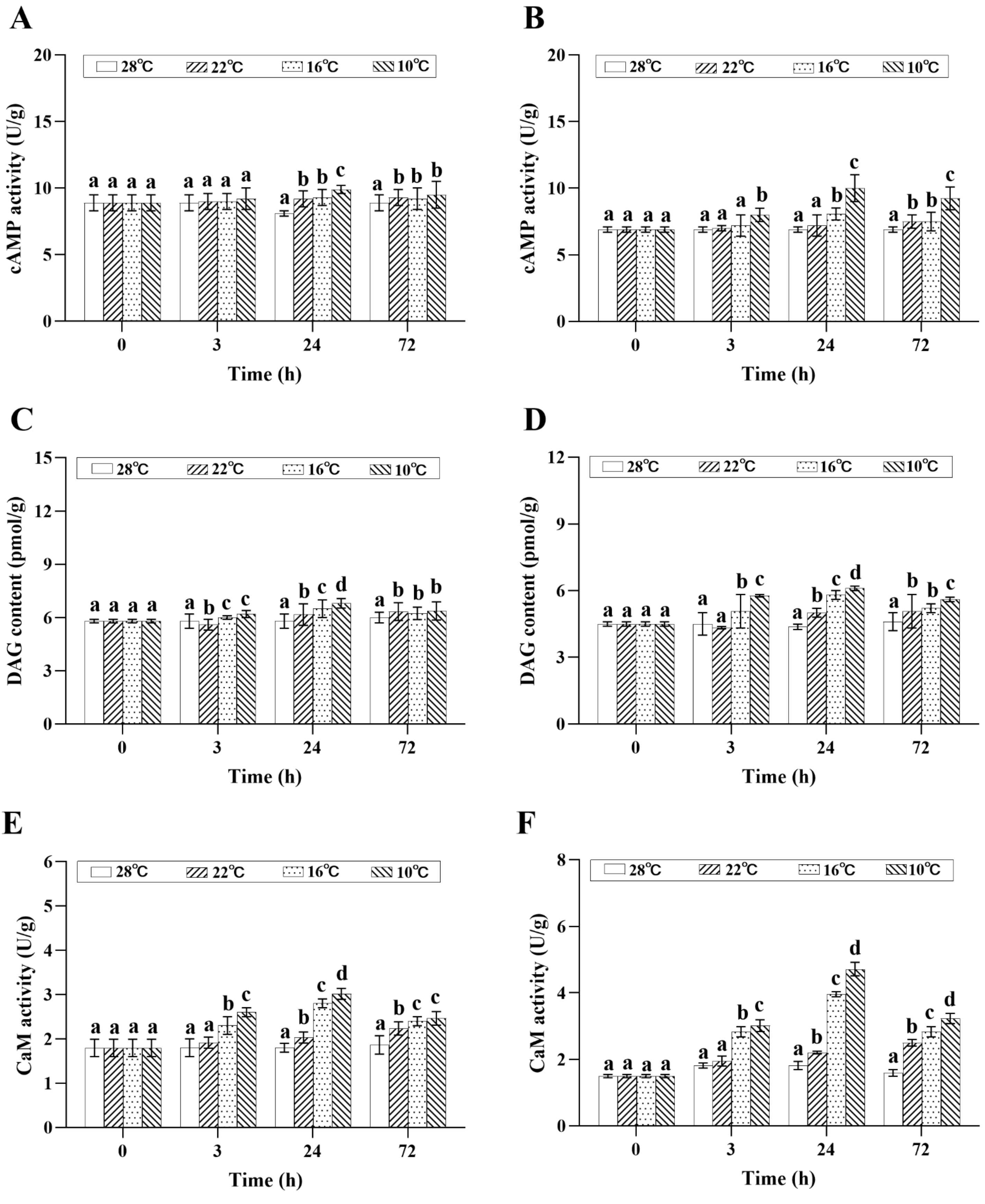

A
B
C
D
E
F
28℃
22℃
16℃
10℃
cAMP activity (U/g)
DAG content (pmol/g)
CaM activity (U/g)
Time (h)
0
3
24
72

Figure. S5 Changes in cAMP, DAG, and CaM levels in the gill (A, C, and E) and hepatopancreas (B, D, and F) of *M. japonicus* according to the time of low temperature stress.

Figure S6

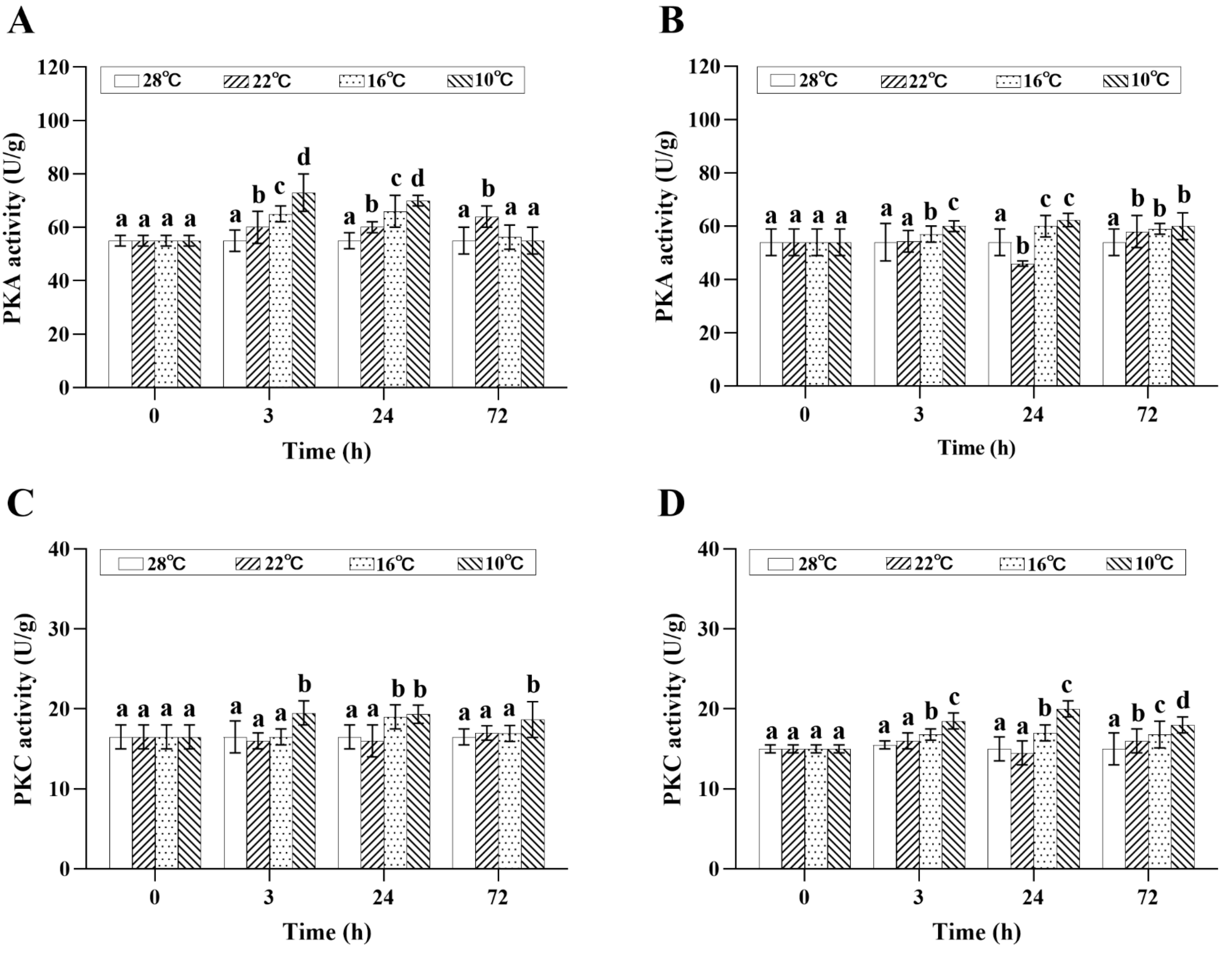

Figure. S6 Changes in PKA and PKC levels the in gill (A, C) and hepatopancreas (B, D) of *M. japonicus* according to the time of low temperature stress.

Figure S7

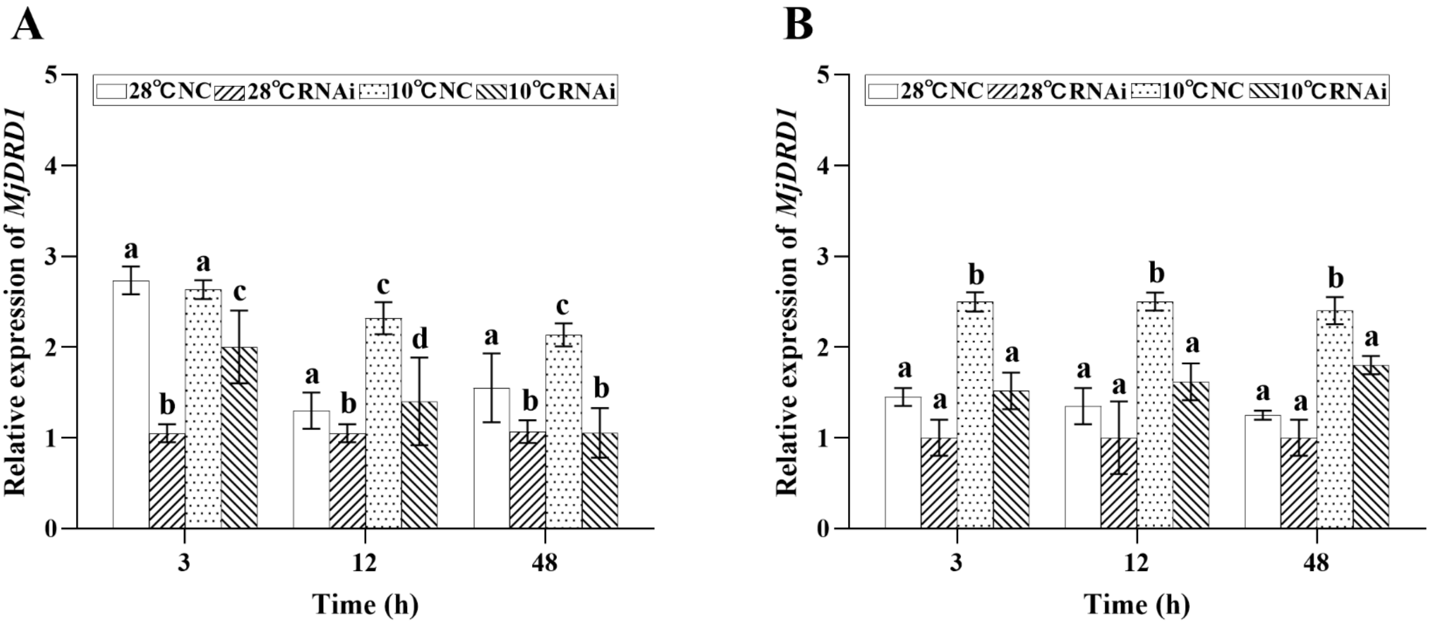

Figure. S7 Changes to the relative expression of *MjDAD1* gene in the *M. japonicus* gill (A) and hepatopancreas (B) after *MjDAD1* interference.
